# The Incidence of Adjacent Segment Degeneration after Cervical Disc Arthroplasty (CDA): A Meta Analysis of Randomized Controlled Trials

**DOI:** 10.1371/journal.pone.0035032

**Published:** 2012-04-25

**Authors:** Baohui Yang, Haopeng Li, Ting Zhang, Xijing He, Siyue Xu

**Affiliations:** Department of Orthopaedic Surgery, The 2nd Affiliated Hospital of Medcal College, Xi'an Jiaotong University, Xi'an, Shaanxi Province, P.R. China; University of Tübingen, Germany

## Abstract

**Background:**

Cervical disc arthroplasty is being used as an alternative degenerative disc disease treatment with fusion of the cervical spine in order to preserve motion. However, whether replacement arthoplasty in the spine achieves its primary patient centered objective of lowering the frequency of adjacent segment degeneration is not verified yet.

**Methodology:**

We conducted a meta-analysis according to the guidelines of the Cochrane Collaboration using databases including PubMed, Cochrane Central Register of Controlled Trials and Embase. The inclusion criteria were: 1) Randomized, controlled study of degenerative disc disease of the cervical spine involving single segment or double segments using Cervical disc arthroplasty (CDA) with anterior cervical discectomy and fusion (ACDF) as controls; 2) A minimum of two-year follow-up using imaging and clinical analyses; 3) Definite diagnostic evidences for “adjacent segment degeneration” and “adjacent segment disease”; 4) At least a minimum of 30 patients per population. Two authors independently selected trials; assessed methodological quality, extracted data and the results were pooled.

**Results:**

No study has specifically compared the results of adjacent segment degenerative; Two papers describing 140 patients with 162 symptomatic cervical segment disorders and compared the rate of postoperative adjacent segment disease development between CDA and ACDF treatments, three publications describing the rate of adjacent-segment surgery including 1273 patients with symptomatic cervical segments. The result of the meta-analysis indicates that there were fewer the rate of adjacent segment disease and the rate for adjacent-segment surgery comparing CDA with ACDF, but the difference was not statistically significant.

**Conclusions:**

Based on available evidence, it cannot be concluded, that CDA can significantly reduce the postoperative rate of the adjacent segment degenerative and adjacent segment disease. However, due to some limitations, the results of this meta-analysis should be cautiously accepted, and further studies are needed.

## Introduction

Cervical spondylosis is a common pathological condition that affects the adult spine, and is the most frequent cause of cervical radiculopathy and myelopathy in older patients. Anterior cervical discectomy and fusion is regarded as a gold standard treatment for degenerative cervical spine disease. It was reported that this treatment provide greater than 90% likelihood of relief of radicular complaints and stabilization/improvement of myelopathic findings [1], [2]. Fusion of the cervical spine has biomechanical consequences. Loss of mobility at one functional spinal unit increases the load sustained by the remaining units [3]–[4]. Anterior cervical fusion has been shown to be associated with the development of new degenerative changes at levels immediately adjacent to the fused segments [5]–[10]. However, the frequency, cause, and clinical significance of these adjacent segment changes remain controversial. Different rates of adjacent segment degeneration have been reported in the literature [1], [5], [7]–[9], [10]–[12] and have varied according to the definition of adjacent segment degeneration.

In recent years, to avoid confusion, Hilibrand et al [13] classified degeneration of adjacent segments into “adjacent segment degeneration” and “adjacent segment disease”. The term “adjacent segment degeneration” is used to describe radiographic changes seen at the adjacent discs compared to the results before the spinal fusion procedure that do not necessarily correlate with any clinical findings. On the other hand, the term “adjacent segment disease” is used to refer to the development of new clinical symptoms that correspond to radiographic adjacent disc changes after a previous spinal fusion.

Many systemic articles reported that fusion might be an important factor of causing (vidence level IV) adjacent segment degeneration and adjacent segment disease. Baba et al. assessed over 100 patients undergoing anterior cervical fusion for cervical myelopathy with an average of 8.5 years of follow-up [5]. The authors observed that 25% of these patients subsequently developed new spinal canal stenoses above the previously fused segments. Similarly, Gore and Sepic [14] observed new spondylosis in 25% of 121 patients and progression of preexisting spondylosis in another 25% of patients who had previously undergone anterior cervical fusion with an average follow-up of 5 years. Bohlman et al. reviewed 122 patients after anterior discectomy and fusion for radiculopathy with an average of 6 years of follow up and found that 9% of these patients went on to develop adjacent segment diseases requiring additional surgery [1]. In addition, Williams et al. found that 17% of their 60 patients undergoing anterior cervical decompression and fusion developed an adjacent segment disease and needed an additional surgery with an average follow-up of 4.5 years [15].

It was hypothesized that fusion can cause increased stress at the adjacent segments and accelerate their degeneration. Therefore, the technique of a non-fusion operation was developed for preserving movement functions and decreasing physical strain stress of the adjacent segments. In the first decade of the 21st century, non-fusion operation such as CDA or cervical disc replacement has been greatly improved. In theory, CDA should decrease the likelihood of developing adjacent segment degeneration and segment breakdown by maintaining normal disc kinematics. In biomechanical cadaveric studies, cervical arthroplasty has also been shown to maintain motion and mechanics within physiologic ranges at the index segment and decrease stresses on adjacent segments [16], [17].

However, few clinical studies have specifically aimed to evaluate adjacent segment degeneration after CDA; only two articles mentioning adjacent segment diseases and showed that total disc arthroplasty did not affect the incidence of adjacent segment disease in the cervical spine(level IIa–b) [18], [19].

Whether replacement arthoplasty in the spine will achieve its primary patient centered goals with improved outcomes and less adjacent segment degeneration remains an open question. In order to clarify this debate further, we searched available medical databases for published trials and performed a meta-analysis to evaluate the role of cervical arthroplasty in reducing adjacent-disc segment degeneration.

## Materials and Methods

We conducted a meta-analysis using the guidelines of the Cochrane Collaboration [20], and our findings are reported according to the Quality of Reporting of Meta-analyses statement [21].

### Considering criteria for this meta analysis study

#### Types of studies

Due to the availability of RCTs comparing CDA with ACDF, only randomized trials were evaluated. In theory, to avoid repetition of data, single-center studies that have been described as part of multicenter studies, but when outcome data were not repeated, we also adopted them as a study. Garrido et al [22] was a single-center study and was included in Rick et al [23] but they mentioned adjacent segment diseases, whereas Rick et al did not. Publications with fewer than 30 patients per group may not reveal a real difference in the distribution of outcomes, and thus were discarded. To avoid confusion with reoperation rates for other conditions, only studies with at least 2 years of follow-up were included in our study.

#### Types of Participants

The 2 treatment groups were similar demographically, and there were no statistically significant differences (p<0.05) with respect to the variables of age, sex, smoking, or work status. Patient in both groups had failed active conservative management for at least 6 months.

#### Types of interventions

Jawahar et al [18] highlighted the calculated non statistical significance of one vs two segment degenerative disc diseases (DDD) for developing adjacent segment degenerative diseases. Therefore we compared the results of surgical treatment of one– and/or two-segment DDD treated by ACDF or CDA.; Among these studies, each group selected artificial disc prosthesis type almost all not identical, and in one study which even use a variety of artificial intervertebral disc, therefore, we are unable to prosthesis which is constrained, semi constrained or unconstrained detailed classification.

#### Types of Outcomes Studied

In our study, we used Hilibrand' [13]definitions to classify degeneration of adjacent segments into “adjacent segment degeneration” and “adjacent segment disease” Radiological assessment of adjacent segment degeneration: Radiological change of adjacent segment on serial plain radiographs before and after cervical ADR was investigated. And included new formation or enlargement of anterior osteophyte and new or increase of ALL calcification documented by serial plain radiographs. New narrowing of disk space and disk degeneration by MRI-defined degeneration category [24] as the radiological evidence of adjacent segment degeneration [25].

The objective of this manuscript is to analyze if CDA lowers degeneration in adjacent segment and therefore the outcomes to be studied are the presence of adjacent segment degeneration and adjacent segment disease. Although surgery of adjacent segments is not synonymous with adjacent segment disease, it indirectly reflects the rate of adjacent segment degenerative diseases, and we used it also as a secondary evaluation standard.

### Search Strategy

The following search terms were used: cervical spine OR cervical spine arthroplasty OR cervical spine AND artificial disc OR cervical spine arthrodesis OR cervical spine fusion AND “randomized controlled trials”. Because the publication of the first study that described a commercially available CDA device was in 2002 [26], our literature publication time range was defined from December 2001 to September 2011 (last searched September 2011; see Table S1). The databases included PubMed, Cochrane Central Register of Controlled Trials, and Embase with no language restriction. In addition, we also performed hand-searching of informations in the Orthopedics China Biological Medicine Database.

### Data collection and analysis

#### Inclusion and exclusion criteria

The inclusion criteria were: 1) Randomized, controlled study of degenerative disc disease of the cervical spine involving single segment or double segments using CDA with anterior cervical discectomy and fusion (ACDF) as controls; 2) A minimum of two-year follow-up using imaging and clinical analyses; 3) Definite diagnostic evidences for “adjacent segment degeneration” and “adjacent segment disease”; 4) At least a minimum of 30 patients per population.

Exclusion criteria were: 1) case reports; 2) reviews; 3) patients with cervical spine disease involving more than three segments.( Excluded studies and main reason; see Table S2)

#### Selection of studies

Both authors (BaoHui Yang and HaoPeng Li) assessed potentially eligible trials for inclusion with any disagreement being resolved through discussion. Titles of journals, names of authors or supporting institutions were not masked at any stage.

#### Data extraction and management

Data were extracted independently by both authors using piloted forms. The data included the general characteristics of each study and the outcomes measured. General characteristics included study design, first author, year of publication, sample size, interventions and various types of artificial total disc replacements. The outcomes measured included: the rate of postoperative development of adjacent segment degenerative or diseases and the rate of adjacent-segment surgery. Discrepancies were resolved through discussion.

### Assessment of bias inclusion risk in the study

To avoid inherent problems with scale validity [20], we did not use quality scale or checklists. We assessed the methodological quality as described by the Cochrane Reviews Handbook 5.0.2 [21], (Table 1. Methodological quality assessment scheme), The studies were classified into A: low risk of bias and each of the criteria was appropriate, B: medium risk of bias and most of the criteria were appropriate, and C: high risk of bias and most of the criteria were not appropriate. (methodological domain assessment for each including study; see: Table S3).

**Table 1:** The Cochrane Collaboration's tool for assessing risk of bias.

Domain	Description	Review authors' judgement
**Sequence generation**	Describe the method used to generate the allocation sequence in sufficient detail to allow an assessment of whether it should produce comparable groups.	Was the allocation sequence adequately generated? (Yes/No/Unclear)
**Allocation concealment.**	Describe the method used to conceal the allocation sequence in sufficient detail to determine whether intervention allocations could have been foreseen in advance of, or during, enrolment	Was allocation adequately concealed?(Yes/No/Unclear)
**Blinding of participants, personnel and outcome**	Describe all measures used, if any, to blind study participants and personnel from knowledge of which intervention a participant received. Provide any information relating to whether the intended blinding was effective.	Was knowledge of the allocated intervention adequately prevented during the study? (Yes/No/Unclear)
**Incomplete outcome data**	Describe the completeness of outcome data for each main outcome, including attrition and exclusions from the analysis. State whether attrition and exclusions were reported, the numbers in each intervention group (compared with total randomized participants), reasons for attrition/exclusions where reported, and any re-inclusions in analyses performed by the review authors.	Were incomplete outcome data adequately addressed?(Yes/No/Unclear)
**Selective outcome reporting.**	State how the possibility of selective outcome reporting was examined by the review authors, and what was found.	Are reports of the study free of suggestion of selective outcome reporting? (Yes/No/Unclear)
**Other sources of bias.**	State any important concerns about bias not addressed in the other domains in the tool. If particular questions/entries were pre-specified in the review's protocol, responses should be provided for each question/entry.	Was the study apparently free of other problems that could put it at a high risk of bias? (Yes/No/Unclear)

### Measures of treatment effect

Only dichotomous outcomes were mentioned in our study, so the odds ratio (OR) or risk ratios and 95% confidence intervals were calculated for outcomes.

### Assessment of heterogeneity

The heterogeneity test P values revealed by the forest plot were used to determine the heterogeneity of the included studies. I^2^ was used to estimate the size of the heterogeneity. I^2^>50% indicated considerable heterogeneity among the included studies.

### Data synthesis

Results of comparable groups of trials were pooled using the fixed-effects model and 95% confidence intervals. When there was a clear or significant heterogeneity, we viewed the results of a random effect model, but in cases where the outcome measures were clearly different we opted not to pool the data.

### Subgroup analysis and investigation of heterogeneity

If heterogeneity was determined using the above methods, the causes of heterogeneity was first analyzed and then subjected to sub-group treatment. If such treatment still could not eliminate the statistical heterogeneity, a random effect model was used for the combined analysis of the studies, in case they showed clinical consistency.

### Sensitivity analysis

Reanalyzing the data using different statistical approaches (e.g. using a random effect model instead of a fixed effects model or vice versa) was used for the sensitivity analysis in our meta analysis.

## Results

### Description of studies

**Figure 1:**
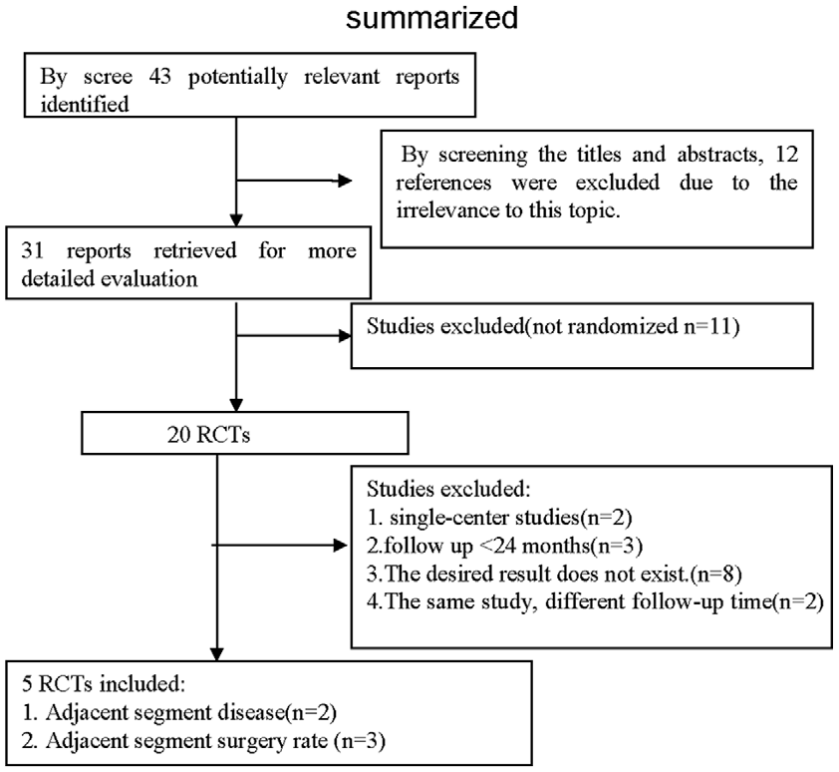
The process of identifying relevant studies is summarized.

The process of identifying relevant studies is summarized in Fig. 1. From the selected databases, 43 references were obtained. By screening the titles and abstracts, 12 references were excluded due to the irrelevance to this topic. In 31 potentially relevant references, 26 references were omitted according to the conditions. 5 randomized control trials were eventually included in the meta-analysis [18], [19], [22], [23], [27]. Jawahar et al. [18] used three devices, including Kineflex-C (SpinalMotion Inc., Mountain View, CA, USA), Mobi-C (LDR spine, Austin, TX, USA), and Advent Cervical Disc (Blackstone Inc., Parsippany, NJ, USA). Garrido et al. [22] used The Bryan Device. Burkus et al.[19] used Prestige disc prosthesis and Coric et al.[27] used Kineflex|C artificial disc placement. 22 patients received operations of two segmental lesions and the rest for a single segmental lesion. All included studies stated that the operations were performed on C3–C7 but did not give the specific location of the treated segments. The study characteristics of these 5 studies are shown in Tables 2.

**Table 2:** Characteristics of included randomized controlled trials.

Study	Methods	Participants	Interventions	Outcome
Garrido BJ (US)	R:1:1rati(randomized)C: unclearB: unclear L: 9/47(48 months)	47 Patients; 30males,17 females;One level = 47	CAD = 21; ACDF = 26; CAD(Bryan Cervical Disc)	Adjacent segment disease: CAD = 1, CAD = 3.
Jawahar A (US)	R: computer-generated C: unclear B: patients L: 28/93(48 months)	93 Patients; 56 males,37 females;One level = 71;Two level = 22	CAD = 59; ACDF = 34; CAD (Kineffiex-C, Mobi-C, Advent Cervical Disc)	Adjacent segment disease: CAD = 6, CAD = 5.
DomagojCoric (US)	R:1:1rati(randomized)C: unclear B: unclear L: 35/269(48 months)	269 Patients;One level = 269;	CAD = 136; ACDF = 133; CAD (Kineffiex-C)	Reoperations were required for adjacent-segment disease: CAD = 1; ACDF = 5;
Rick C(US)	R:1:1rati(randomized)C: unclear B: investigators and patients L:154/463(48 months)	463 Patients; 223 males,240 females;One level = 463	CAD = 242; ACDF = 221; CAD (Bryan Cervical Disc)	Reoperations were required for adjacent-segment disease CAD = 9; ACDF = 9;
Burkus JK (US)	R:randomization number C:no B: unclear L: 270/541(60 months)	541 Patients; 250 males,291 females;One level = 541	CAD = 276; ACDF = 265; CAD (PRESTIGE ST Cervical Disc System)	Reoperations were required for adjacent-segment disease: CAD = 11; ACDF = 16;

R randomization, C concealment of allocation, B blinding, L losses to follow-up.

### Clinical Heterogeneity

Although patients had similar characteristics and definite inclusion/exclusion criteria, there was a considerable clinical heterogeneity between the studies. For example, various types of artificial total discs were used in the 5 trials; Rick et al. [27] and Garrido et al [22] performed the Bryan Cervical Disc replacement in contrast to ACDF and Kineflex-C, ProDisc-C treatment of others, with different cervical disc replacement devices used. The described arthroplasty devices have different mechanical properties and a device can be constrained, semi constrained or unconstrained in a description of the motion that the device allows relative to other devices and normal motion. These different implant types result in different instantaneous centers of rotation and therefore mimic the natural situation to varying degrees and also may affected the incidence of adjacent segment degeneration [28].

Hongyan et al. found that C5-6 and C6-7 fusion were more susceptible to adjacent segment degeneration, while C2-3 and C7-T1 were not [29]. Unfortunately, all included studies stated that the operation was performed in C3–C7 but did not give the specific location of the treated segments.

Jawahar et al includes one– and/or two– symptomatic cervical segments DDD, the remaining studies are one-symptomatic cervical segment DDD [18]. It was reported that multi segment fusion can increase the degeneration of adjacent segments. However, Hilibrand et al [9] reviewed 374 patients who had undergone anterior cervical fusion with a 21-year follow-up duration an found that the risk of a new disease at an adjacent segment was significantly lower following a multi-segment fusion than it was following a single-segment fusion, and the preoperative radiographic evidence of degeneration at adjacent segments appear to be the greatest risk factors for postoperative adjacent segment disease.

The study follow-up period varied from 48–60 months. Because cervical spondylosis is a virtually inevitable consequence of the normal aging process, it is difficult to ascertain whether adjacent segment changes are a cause of the index procedure, or merely a later manifestation of the initial disease process. all of them might result in potential bias.

### Risk of bias inclusion in the studies

**Figure 2:**
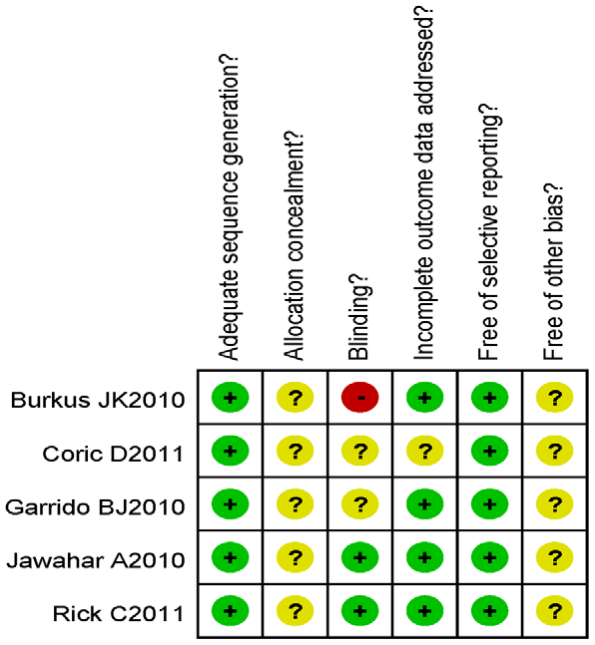
Risk of bias summary. Review authors' judgments about each risk of bias item for each included study. + is “yes”, − is “no”,? is “unclear”.

Fig. 2 provides a summary of methodological domain assessments for each including study. Overall, the methodological quality of all trials was found to be medium risk of bias. The randomization technique was mentioned in all 5 trials, including computer-generated, randomization number and 1∶1 ratio, but no trials mentioned allocation concealment. Blinding is rarely used in orthopedic surgery trials and only one study was single-blinded for the patients [18], whereas another study was double-blinded to the observers, patient investigators and patients [22], what might have resulted in a potential selection bias. In addition the various types of artificial total disc used in 5 trials might result in performance bias. Although there were lost follow-up phenomenon in five studies, missing outcome data balanced in numbers across intervention groups, with similar reasons for missing data across the groups, resulting in a low attrition bias risk.

In addition unpublished negative results might have biased the results toward successful treatments and because 5 articles came from the United States a publication bias towards particular American treatments, postoperative behaviors and clinical cares e.g. duration of hospitalization cannot be excluded.

### Outcomes Measured

No study has specifically compared the results of arthroplasty with the results of fusion with respect to the rate of postoperative development of adjacent segment degenerative.

**Figure 3:**
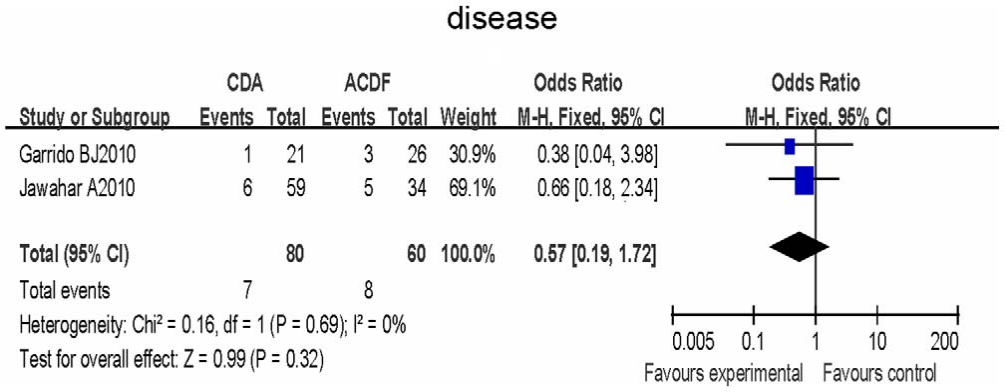
Forest plot of comparison Adjacent segment disease. The result indicates that there were fewer Adjacent segment disease comparing CDA to ACDF, but the difference was not statistically significant.

Two trials reported the adjacent segment disease and were included in the meta-analysis and there was no statistical heterogeneity between all studies (I^2^ = 0%). Using the fixed-effects model, the rate of adjacent segment disease was fewer in CDA (8.8%) compared to ACDF (13%), but the difference was not statistically significant, (RR, 0.57; 95% CI, 0.19,1.72; P = 0.32)( Fig. 3).

**Figure 4:**
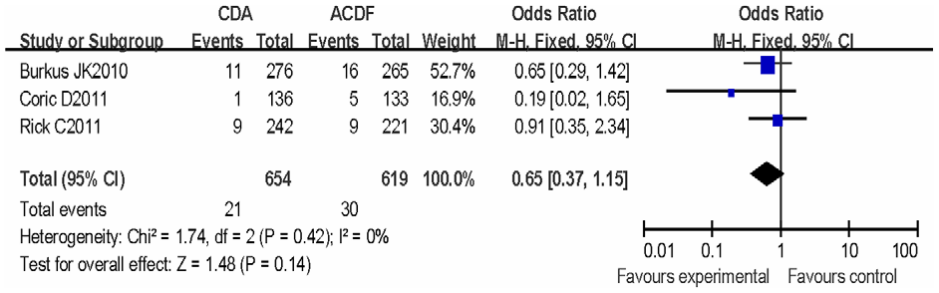
Forest plot of comparison Adjacent segment reoperations. The result indicates that there were fewer there were fewer Adjacent segment reoperations comparing CDA to ACDF, but the difference was also not statistically significant.

Three trials reported reoperations were required for adjacent-segment diseases, and there was no evidence of statistical heterogeneity between all studies (I^2^ = 0%). Using the fixed-effects model, the rate of adjacent-segment surgery was fewer in CDA (3.21%) compared to ACDF (4.84%), but the difference was also not statistically significant, (RR, 0.65; 95% CI, 0.37,1.15; P = 0.14)( Fig. 4).

## Discussion

Several published series have reported satisfactory clinical and radiological outcomes of the ACDF procedure in providing symptomatic relief and restoring neurological functions [30], [31]. However, with prolonged follow-up time and increased number of cases, the adjacent segment degeneration caused the attention of researcher after fusion. They reported that there are a lot of factors influencing adjacent segment degeneration: age, sex, fusion segment, adjacent segment difference, fusion and fixation method as well as others. A lot of literature about clinical and experimental studies reported their observation of anterior cervical dynamic changes and biomechanical changes after fusion surgery, and summarized the pathological cause factors and mechanisms of adjacent segment degeneration. Jason et al. suggested that maintaining normal physiological cervical vertebra curvature after ACDF operation should be considered as one of the important factors that affect the clinical efficacy of a cervical treatment [32]. Others also believe that the physiological curvature of the cervical vertebra is an important factor for developing accelerated adjacent segment degeneration. Katsuura [31] observed a number of traditional ACDF patients with a mean follow-up time of 9.8 years and found that 43% of these patients showed cervical kyphosis or C shape abnormalities and the occurrence of adjacent segment degeneration. Takeshima [33] analyzed the reason and suggested that the cervical dynamic change may increase the adjacent intervertebral stress and lead to accelerated degeneration of adjacent segments. Further, Jason et al [32] studied the up and down adjacent intervertebral disc biomechanics changes after C5∼6 single segment screw plate fixation. They proposed that degeneration is a result of adjacent segment degeneration after fusion operation in combination with normal physiological degeneration.

However, cervical vertebra diseases certainly involve the age factor. It is argued that adjacent segment degeneration results from the natural progression of degenerative disc diseases. In a comparative radiographic study, Herkowitz et al [34] studied 44 patients with 4.5 years follow-up who had been randomized to anterior cervical discectomy and fusion or posterior foraminotomy without fusion for the treatment of cervical radiculopathy. Among the group undergoing anterior fusion, 41% developed adjacent segment degeneration. Surprisingly, however, 50% of the patients undergoing posterior foraminotomy without fusion had evidence of adjacent level degeneration. Once again, there was no correlation between the development of adjacent segment degeneration and the onset of new clinical symptoms referable to those radiographic changes. Hilibrand and Robbins results implied that adjacent segment disease was indeed a common problem but may reflect the natural history of the underlying cervical spondylosis [9], [14]. Recently, one article reported that they divided anterior fusion patients in two groups according to the age. There were 38 patients younger than 50 years, and 49 patients older than 50 years. Regarding the number of cases that showed new degenerative developments, no significant difference was observed between these two groups in terms of the development of new radiological degenerative changes (p = 0.83). The percentage of patients that developed adjacent segment disease was greater in older group (6%) than in the younger group (2%) [35].

There are many and complicated reasons for developing adjacent segment degeneration after ACDF and CDA, mainly including the increased adjacent vertebral sagittal activity [36], the fusion segment number [9], the segment locations [9], segmental kyphosis operation [33], and the influence of each factor on the other (1). ACDF may increase the stress of fused adjacent segments, which is the reason of causing adjacent segment degeneration [37], but if ACDF operation can preserve or even reconstruct segmental lordosis, it will reduce the incidence of adjacent segment degeneration [33]; after adjacent segment degeneration operation segmental kyphosis may appear during maintaining the original stress [38], [39] and induce the incidence of adjacent segment degeneration. Further, the activity of the adjacent segment after ACDF operation is changing with time and [9] section. Anyway, about the method for conserving the spine biomechanics there still exist many controversial opinions, whether treatment should control the displacement or control torque, and it is difficult to fully comply with the actual movement condition of the cervical spine in the human body.

Compared with cervical fusion, disc replacement offers the theoretical biomechanical advantage of preservation of motion, reducing stresses at the adjacent discs, but few clinical studies have specifically aimed to evaluate ASD after CDA or fusion. We only found two articles mentioning adjacent-segment diseases, and in addition, we found 3 articles, which mentioned reoperations required for adjacent-segment diseases. A recent article [7] found that there were no significant differences in the rates of adjacent segment degeneration between cervical fusion and cervical disc replacement groups, with the rate of adjacent segment degeneration higher in patients with lumbar degeneration.

Our meta-analysis showed less postoperative adjacent segment disease incidence in CDA (8.8%) compared to ACDF (13%), but not with statistical significance also for the requirement of reoperation due to adjacent segment degeneration. We suggest that adjacent segment degeneration is affected by the patient individuality and not only by fusion.

Our findings are mainly limited by the quality and number of included studies. First, the number of articles may be insufficient and in the evaluation we incorporated only 5 studies, what might have led to an insufficient significant effectiveness. Second, the low number of included studies limited our assessment of a potential publication bias which cannot be excluded e.g. due to unpublished negative research results. Therefore, publication bias may exist, which might have resulted in the overestimation of the intervention effectiveness. Third, the methodological quality of all trials was found to be poor. Due to these limitations, the combined results of this meta-analysis should be cautiously accepted, and more independent high-quality RCTs with effectiveness analyses is needed. Additionally, in our study we did not detailed analyze prosthesis separately, what may influence the outcome of the analysis.

Although biomechanical studies have shown that fusion leads to increased stress of adjacent segments, whether adjacent segment degeneration is a natural consequence of aging or a complication of fusion remains controversial. Based on available evidence, it cannot be significantly concluded, that cervical disc replacement can reduce the rate of postoperative development of adjacent segment degenerative. However, due to some limitations, the results of this meta-analysis should be cautiously accepted, and high-quality RCTs with long term follow-up and large sample size are needed.

## Supporting Information

Table S1 **Cochrane Central Register of Controlled Trials Search Strategy.**
(DOC)

Table S2 **Excluded studies and main reason for exclusion from the analysis.**
(DOC)

Table S3 **Methodological domain assessment for each including study.**
(DOC)
