# Supplementary material for: The Incidence of Adjacent Segment Degeneration after Cervical Disc Arthroplasty (CDA): A Meta Analysis of Randomized Controlled Trials
Source: PLoS One. 2012 Apr 25;7(4):e35032. doi: 10.1371/journal.pone.0035032 (PMC3338823; doi:10.1371/journal.pone.0035032)
Supplement: Table S1 — Cochrane Central Register of Controlled Trials Search Strategy. (DOC) [file pone.0035032.s001.doc]

Table S1 Cochrane Central Register of Controlled Trials Search Strategy

| **ID** | **Search** | **Hits** | **Edit** | **Delete** |
| --- | --- | --- | --- | --- |
| #1 | [cervical spine](http://onlinelibrary.wiley.com/o/cochrane/searchHistory?mode=runquery&qnum=1) | 902 | [edit](javascript:doPopup('/search-web/cochrane/searchHistory?mode=editquery&qnum=1&searchKey=f243b1bd-9d60-4958-897e-080eac1b0ade', 400)) | [delete](http://onlinelibrary.wiley.com/search-web/cochrane/searchHistory?mode=deletequery&qnum=1&uuid=f243b1bd-9d60-4958-897e-080eac1b0ade&searchKey=f243b1bd-9d60-4958-897e-080eac1b0ade) |
| #2 | [(arthroplasty):ti,ab,kw or artifcial disc:ti,ab,kw or (arthrodesis ):ti,ab,kw or (disc arthroplasty):ti,ab,kw in Clinical Trials](http://onlinelibrary.wiley.com/o/cochrane/searchHistory?mode=runquery&qnum=2) | 3103 | [edit](javascript:doPopup('/search-web/cochrane/searchHistory?mode=editquery&qnum=2&searchKey=f243b1bd-9d60-4958-897e-080eac1b0ade', 400)) | [delete](http://onlinelibrary.wiley.com/search-web/cochrane/searchHistory?mode=deletequery&qnum=2&uuid=f243b1bd-9d60-4958-897e-080eac1b0ade&searchKey=f243b1bd-9d60-4958-897e-080eac1b0ade) |
| #3 | [(fusion):ti,ab,kw or (discectomy):ti,ab,kw in Clinical Trials](http://onlinelibrary.wiley.com/o/cochrane/searchHistory?mode=runquery&qnum=3) | 2444 | [edit](javascript:doPopup('/search-web/cochrane/searchHistory?mode=editquery&qnum=3&searchKey=f243b1bd-9d60-4958-897e-080eac1b0ade', 400)) | [delete](http://onlinelibrary.wiley.com/search-web/cochrane/searchHistory?mode=deletequery&qnum=3&uuid=f243b1bd-9d60-4958-897e-080eac1b0ade&searchKey=f243b1bd-9d60-4958-897e-080eac1b0ade) |
| #4 | [(#1 AND #2)](http://onlinelibrary.wiley.com/o/cochrane/searchHistory?mode=runquery&qnum=4) | 47 | [edit](javascript:doPopup('/search-web/cochrane/searchHistory?mode=editquery&qnum=4&searchKey=f243b1bd-9d60-4958-897e-080eac1b0ade', 400)) | [delete](http://onlinelibrary.wiley.com/search-web/cochrane/searchHistory?mode=deletequery&qnum=4&uuid=f243b1bd-9d60-4958-897e-080eac1b0ade&searchKey=f243b1bd-9d60-4958-897e-080eac1b0ade) |
| #5 | [(#1 AND #3)](http://onlinelibrary.wiley.com/o/cochrane/searchHistory?mode=runquery&qnum=5) | 196 | [edit](javascript:doPopup('/search-web/cochrane/searchHistory?mode=editquery&qnum=5&searchKey=f243b1bd-9d60-4958-897e-080eac1b0ade', 400)) | [delete](http://onlinelibrary.wiley.com/search-web/cochrane/searchHistory?mode=deletequery&qnum=5&uuid=f243b1bd-9d60-4958-897e-080eac1b0ade&searchKey=f243b1bd-9d60-4958-897e-080eac1b0ade) |
| #6 | [(#4 AND #5)](http://onlinelibrary.wiley.com/o/cochrane/searchHistory?mode=runquery&qnum=6) | 39 | [edit](javascript:doPopup('/search-web/cochrane/searchHistory?mode=editquery&qnum=6&searchKey=f243b1bd-9d60-4958-897e-080eac1b0ade', 400)) | [delete](http://onlinelibrary.wiley.com/search-web/cochrane/searchHistory?mode=deletequery&qnum=6&uuid=f243b1bd-9d60-4958-897e-080eac1b0ade&searchKey=f243b1bd-9d60-4958-897e-080eac1b0ade) |
