# Supplementary material for: The Incidence of Adjacent Segment Degeneration after Cervical Disc Arthroplasty (CDA): A Meta Analysis of Randomized Controlled Trials
Source: PLoS One. 2012 Apr 25;7(4):e35032. doi: 10.1371/journal.pone.0035032 (PMC3338823; doi:10.1371/journal.pone.0035032)
Supplement: Table S2 — Excluded studies and main reason for exclusion from the analysis. (DOC) [file pone.0035032.s002.doc]

Table S2. Excluded studies and main reason for exclusion from the analysis

| Year | 1st Author | Exclusion Reason |
| --- | --- | --- |
| 2007 | Chang UK | cadaveric study |
| 2011 | Park DK | kinematics study |
| 2010 | Menzin J | A Health-economic Assessment |
| 2006 | Laxer EB | cadaveric study |
| 2007 | Chang UK | range of motion study |
| 2008 | Rousseau MA | kinematic study |
| 2008 | Zhang X | mechanical study |
| 2009 | Yang CW | imaging study |
| 2009 | Lin CY | stress analysis |
| 2007 | Sekhon LH | MR imaging study |
| 2007 | Rabin D | kinematics study |
| 2008 | Sasso RC | Kinematics study |
| 2005 | Leung C | not randomized |
| 2004 | Anderson PA | not randomized |
| 2002 | Bryan VE Jr | not randomized |
| 2002 | Goffin J | not randomized |
| 2004 | Robertson JT | not randomized |
| 2009 | Bohlman HH | not randomized |
| 2009 | Peng CW | not randomized |
| 2009 | Röhl K | not randomized |
| 2008 | Sukhomel P | not randomized |
| 2006 | Mehren C | not randomized |
| 2005 | Bertagnoli R | not randomized |
| 2010 | McAfee PC | The desired result does not exist |
| 2009 | Kelly MP | The desired result does not exist |
| 2008 | Wang Y | The desired result does not exist |
| 2010 | Arts MP | The desired result does not exist |
| 2010 | McAfee PC | The desired result does not exist |
| 2009 | Frank M. | The desired result does not exist |
| 2009 | Daniel | The desired result does not exist |
| 2010 | Marzluff J | The desired result does not exist |
| 2007 | Pitzen T | 12 wks FU |
| 2007 | Nabhan A | 1 year FU |
| 2007 | Amit A | 1 year FU |
| 2007 | Sasso RC | part of a major trial |
| 2006 | Coric D | part of major trial |
| 2007  2008 | MUMMANENI  Paul A. | =Burkus2010  =Rick C2011 |
