# Supplementary material for: The Incidence of Adjacent Segment Degeneration after Cervical Disc Arthroplasty (CDA): A Meta Analysis of Randomized Controlled Trials
Source: PLoS One. 2012 Apr 25;7(4):e35032. doi: 10.1371/journal.pone.0035032 (PMC3338823; doi:10.1371/journal.pone.0035032)
Supplement: Table S3 — Methodological domain assessment for each including study. (DOC) [file pone.0035032.s003.doc]

Table S3 methodological domain assessment for each including study

| **Items / Trials** | **Rick C2011** | **Garrido 2010** | **Jawahar2010** | **Burkus2010** | **Domagoj Coric2011** |
| --- | --- | --- | --- | --- | --- |
| **Sequence generation** | Yes | Yes | Yes | Yes | Yes |
| **Allocation concealment.** | ? | ? | ? | ? | ? |
| **Blinding** | Yes | ? | Yes | No | ? |
| **Incomplete outcome data** | Yes | Yes | Yes | Yes | ? |
| **Selective outcome reporting.** | Yes | Yes | Yes | Yes | Yes |
| **Other sources of bias** | ? | ? | ? | ? | ? |
